# Supplementary material for: Alternative pathways to α,β-unsaturated ketones via direct oxidative coupling transformation using Sr-doped LaCoO3 perovskite catalyst
Source: R Soc Open Sci. 2019 Nov 27;6(11):191313. doi: 10.1098/rsos.191313 (PMC6894578; doi:10.1098/rsos.191313)
Supplement: Supporting information [file rsos191313supp1.doc]

**Alternative pathways to α,β-unsaturated ketones** **via direct oxidative coupling transformation using Sr-doped LaCoO_3_ perovskite catalyst**

Khang H. Trinh, Son H. Doan, Tien V. Huynh, Phuong H. Tran, Diep T. N. Pham, Minh-Vien Le, Tung T. Nguyen^*^, Nam T. S. Phan^*^

Faculty of Chemical Engineering, HCMC University of Technology, VNU-HCM,

268 Ly Thuong Kiet, District 10, Ho Chi Minh City, Viet Nam

^*^Email: [tungtn@hcmut.edu.vn](mailto:tungtn@hcmut.edu.vn); [ptsnam@hcmut.edu.vn](mailto:ptsnam@hcmut.edu.vn)

Ph: (+84 8) 38647256 ext. 5681 Fx: (+84 8) 38637504

**Supporting information**

*Materials and instrumentation*

All reagents and starting materials were obtained commercially from Sigma-Aldrich and Merck, and were used as received without any further purification unless otherwise noted. Nitrogen physisorption measurements were conducted using a Micromeritics 2020 volumetric adsorption analyzer system. Samples were pretreated by heating under vacuum at 150 ^o^C for 3 h. X-ray powder diffraction (XRD) patterns were recorded using a Cu Kα radiation source on a D8 Advance Bruker powder diffractometer. Scanning electron microscopy studies were conducted on a JSM-6500F, JEOL Scanning Electron Microscope (SEM).

Gas chromatographic (GC) analyses were performed using a Shimadzu GC 2010-Plus equipped with a flame ionization detector (FID) and an SPB-5 column (length = 30 m, inner diameter = 0.25 mm, and film thickness = 0.25 μm). The temperature program for GC analysis held samples at 100 ^o^C for 1 min; heated them from 100 to 280 ^o^C at 40 ^o^C/min; held them at 280 ^o^C for 4.5 min. Inlet and detector temperatures were set constant at 280 ^o^C. The GC yield was calculated using diphenyl ether as the internal standard.

GC-MS analyses were analyzed on a Shimadzu GCMS-QP2010Ultra with a ZB-5MS column (length = 30 m, inner diameter = 0.25 mm, and film thickness = 0.25 μm). The temperature program for GC-MS analysis held samples at 50 ^o^C for 2 min; heated samples from 50 to 280^o^C at 10 ^o^C/min and held them at 280 ^o^C for 10 min. Inlet temperature was set constant at 280 ^o^C. MS spectra were compared with the spectra gathered in the NIST library. The ^1^H NMR and ^13^C NMR were recorded on Bruker AV 500 spectrometers using residual solvent peak as a reference.

Table S1. Screening of reaction conditions^a^.

| Entry | Temperature (^o^C) | Oxidant | Oxidant amount (equiv.) | Catalyst amount (mol%) | Solvent | Yield^b^ (%) |
| --- | --- | --- | --- | --- | --- | --- |
| 1 | RT | DTBP | 4 | 5 | None | 0 |
| 2 | 80 | DTBP | 4 | 5 | None | 5 |
| 3 | 100 | DTBP | 4 | 5 | None | 43 |
| 4 | 120 | DTBP | 4 | 5 | None | 80 |
| 5 | 140 | DTBP | 4 | 5 | None | 5 |
|  | | | | | | |
| 6 | 120 | None | 4 | 5 | None | 7 |
| 7 | 120 | K_2_S_2_O_8_ | 4 | 5 | None | 60 |
| 8 | 120 | AgNO_3_ | 4 | 5 | None | 12 |
| 9 | 120 | Oxygen | 4 | 5 | None | 9 |
| 10 | 120 | H_2_O_2_ | 4 | 5 | None | 4 |
| 11 | 120 | TBHP/water | 4 | 5 | None | 20 |
| 12 | 120 | TBHP/decane | 4 | 5 | None | 10 |
| 13 | 120 | TBPB | 4 | 5 | None | 64 |
| 14 | 120 | CHP | 4 | 5 | None | 22 |
| 15 | 120 | DTBP | 4 | 5 | None | 80 |
|  | | | | | | |
| 16 | 120 | DTBP | 0 | 5 | None | 5 |
| 17 | 120 | DTBP | 1 | 5 | None | 38 |
| 18 | 120 | DTBP | 2 | 5 | None | 45 |
| 19 | 120 | DTBP | 3 | 5 | None | 58 |
| 20 | 120 | DTBP | 4 | 5 | None | 80 |
|  | | | | | | |
| 21 | 120 | DTBP | 4 | 0 | None | 0 |
| 22 | 120 | DTBP | 4 | 1 | None | 17 |
| 23 | 120 | DTBP | 4 | 3 | None | 55 |
| 24 | 120 | DTBP | 4 | 5 | None | 80 |
| 25 | 120 | DTBP | 4 | 7 | None | 80 |
|  | | | | | | |
| 26 | 120 | DTBP | 4 | 5 | PhCl | 6 |
| 27 | 120 | DTBP | 4 | 5 | NMP | 1 |
| 28 | 120 | DTBP | 4 | 5 | DCB | 7 |
| 29 | 120 | DTBP | 4 | 5 | DMSO | 1 |
| 30 | 120 | DTBP | 4 | 5 | THF | 5 |
| 31 | 120 | DTBP | 4 | 5 | Dioxane | 18 |
| 32 | 120 | DTBP | 4 | 5 | DMF | 1 |
| 33 | 120 | DTBP | 4 | 5 | DMAc | 1 |
| 34 | 120 | DTBP | 4 | 5 | None | 80 |

^a^Reaction conditions: 1,1-diphenylethylene (0.3 mmol); benzaldehyde (1 mL); 18 h; under air. ^b^GC yield. DTBP: di-*tert*-butylperoxide; TBHP/water: *tert*-butyl hydroperoxide in water; TBHP/decade: *tert*-butyl hydroperoxide in decane; TBPB: *tert*-butyl peroxybenzoate; CHP: cumyl hydroperoxide; PhCl: chlorobenzene; NMP: *N*-methyl-2-pyrrolidone; DCB: *o*-dichlorobenzene; DMSO: dimethylsulfoxide, THF: tetrahydrofuran; DMF: dimethylformamide; DMAc: *N*,*N*-dimethylacetamide.

Table S2. The direct oxidative coupling of benzaldehyde with 1,1-diphenylethylene using different catalysts^a^.

| Entry | Homogeneous catalyst | Heterogeneous catalyst | Yield^b^ (%) |
| --- | --- | --- | --- |
| 1 | FeCl_2_ |  | 38 |
| 2 | FeCl_3_ |  | 43 |
| 3 | Fe(NO_3_)_3_ |  | 1 |
| 4 | FeSO_4_ |  | 1 |
| 5 | CuSO_4_ |  | 17 |
| 6 | Cu(NO_3_)_2_ |  | 24 |
| 7 | Ni(NO_3_)_2_ |  | 11 |
| 8 | La(NO_3_)_2_ |  | 1 |
| 9 | Co(NO_3_)_2_ |  | 24 |
| 10 | Sr(NO_3_)_2_ |  | 1 |
| 11 |  | Cu-MOF-74 | 27 |
| 12 |  | Cu_2_(BDC)_2_(DABCO) | 29 |
| 13 |  | Cu(BDC) | 35 |
| 14 |  | Fe_3_(BTC)_2_(NDC) | 1 |
| 15 |  | UiO-66.TFA | 1 |
| 16 |  | La_2_O_3_ | 16 |
| 17 |  | CoO | 28 |
| 18 |  | SrO | 1 |
| 19 |  | LaCoO_3_ | 41 |
| 20 |  | La_0.6_Sr_0.4_CoO_3_ | 80 |

^a^Reaction conditions: 1,1-diphenylethylene (0.3 mmol); benzaldehyde (1 mL); DTBP (1.2 mmol); catalyst (5 mol%); 120 ^o^C; 18 h; under air. ^b^GC yield.


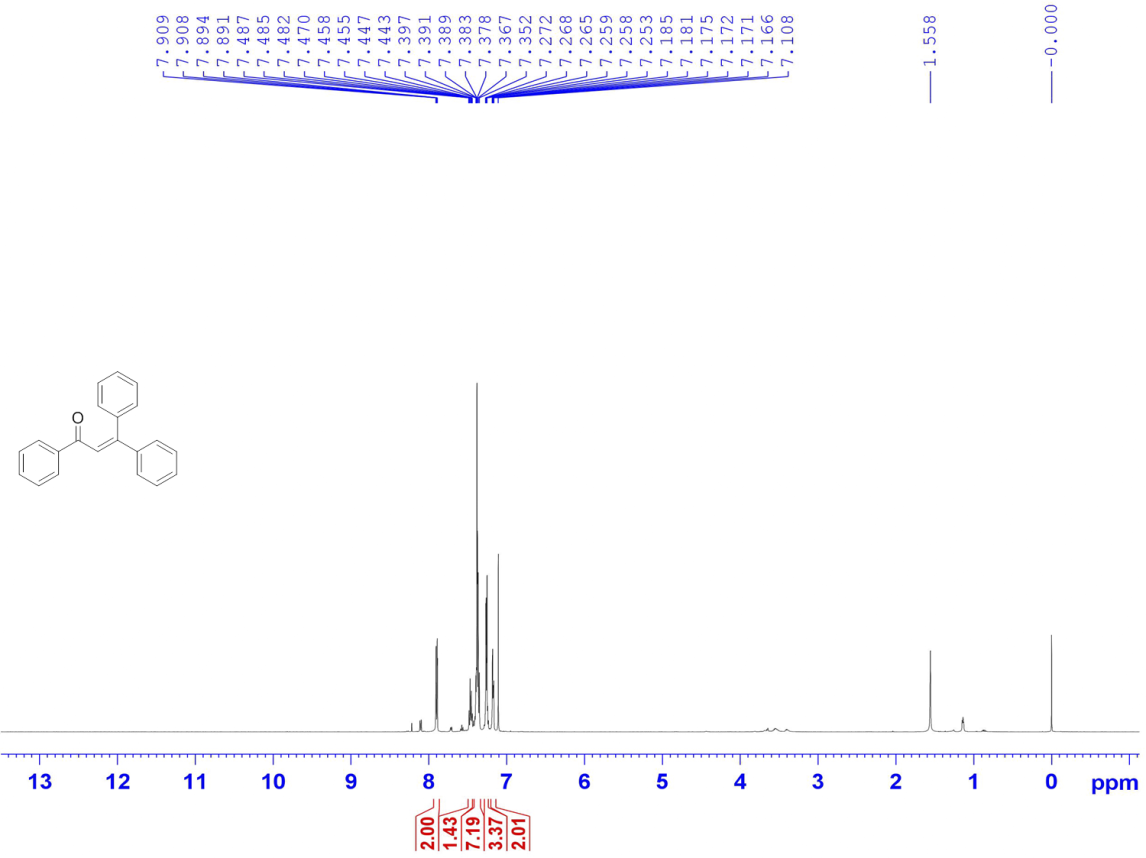


Fig.S3. ^1^H-NMR spectra of 1,3,3-triphenylprop-2-en-1-one.


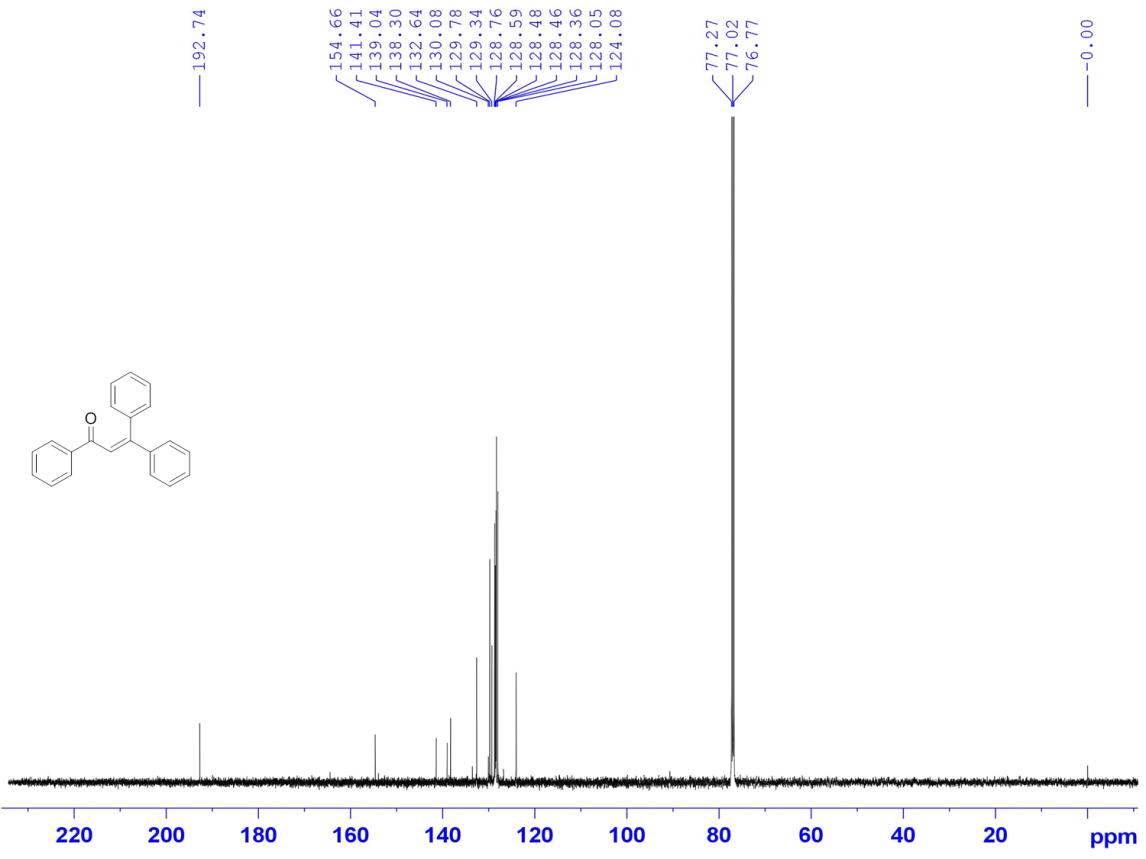


Fig.S4. ^13^C-NMR spectra of 1,3,3-triphenylprop-2-en-1-one.

**Characterization data for 1,3,3-triphenylprop-2-en-1-one**

Prepared as shown in the general experimental procedure and purified on silica gel (230-400 mesh or 37-63 mm, ethyl acetate/hexane = 1:15 (v./v.), TLC silica gel 60 F_254_, R_f_ = 0.4): Light yellow liquid, 75% yield (64 mg). ^1^H-NMR (500 MHz, CDCl_3_) 7.10 (s, 1H), 7.16-7.18 (m, 2H), 7.25-7.27 (m, 3H), 7.35-7.39 (m, 7H), 7.44-7.48 (m, 1H), 7.89 (dd, *J* = 8.0 Hz, 1.0 Hz, 2H). ^13^C NMR (CDCl_3_, 125 MHz) δ(ppm) 124.0, 128.0, 128.3, 128.4, 128.4, 128.5, 128.7, 129.3, 129.7, 130.0, 132.6, 138.3, 139.0, 141.4, 154.6, 192.7.


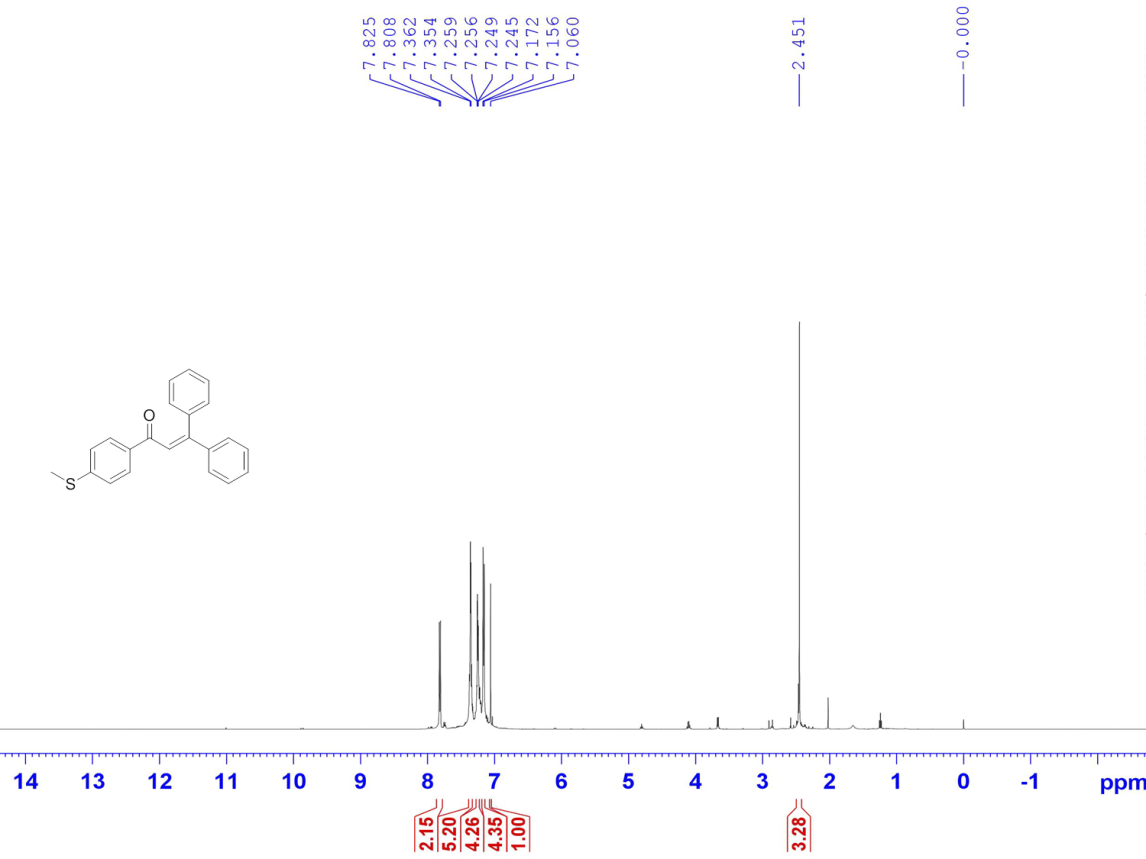


Fig.S5. ^1^H-NMR spectrum of 1-(4-(methylthio)phenyl)-3,3-diphenylprop-2-en-1-one.


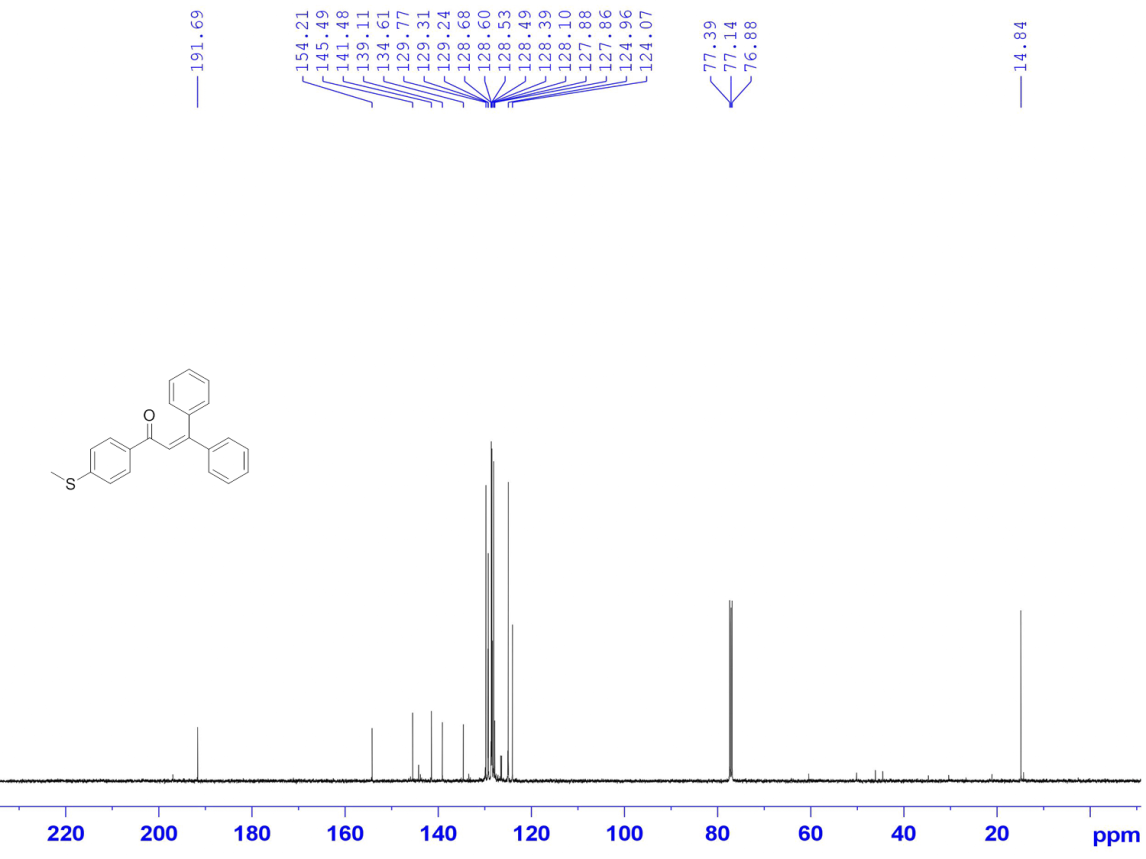


Fig.S6. ^13^C-NMR spectrum of 1-(4-(methylthio)phenyl)-3,3-diphenylprop-2-en-1-one.

**Characterization data for 1-(4-(methylthio)phenyl)-3,3-diphenylprop-2-en-1-one.**

Prepared as shown in the general experimental procedure and purified on silica gel (230-400 mesh or 37-63 μm, ethyl acetate/hexane = 1:20 (v./v.), TLC silica gel 60 F_254_, R_f_ = 0.4): Yellow liquid, 76% yield (76 mg). ^1^H-NMR (500 MHz, CDCl_3_) 2.45 (s, 3H), 7.06 (s, 1H), 7.15 (d, *J* = 8.0 Hz, 4H), 7.24-7.25 (m, 4H), 7.35-7.36 (m, 5H), 7.80 (d, *J* = 8.5 Hz, 2H). ^13^C NMR (CDCl_3_, 125 MHz) δ(ppm) 14.8, 124.0, 124.9, 127.8, 127.8, 128.1, 128.3, 128.4, 128.5, 128.6, 128.6, 129.2, 129.3, 129.7, 134.6, 139.1, 141.4, 145.4, 154.2, 191.6.


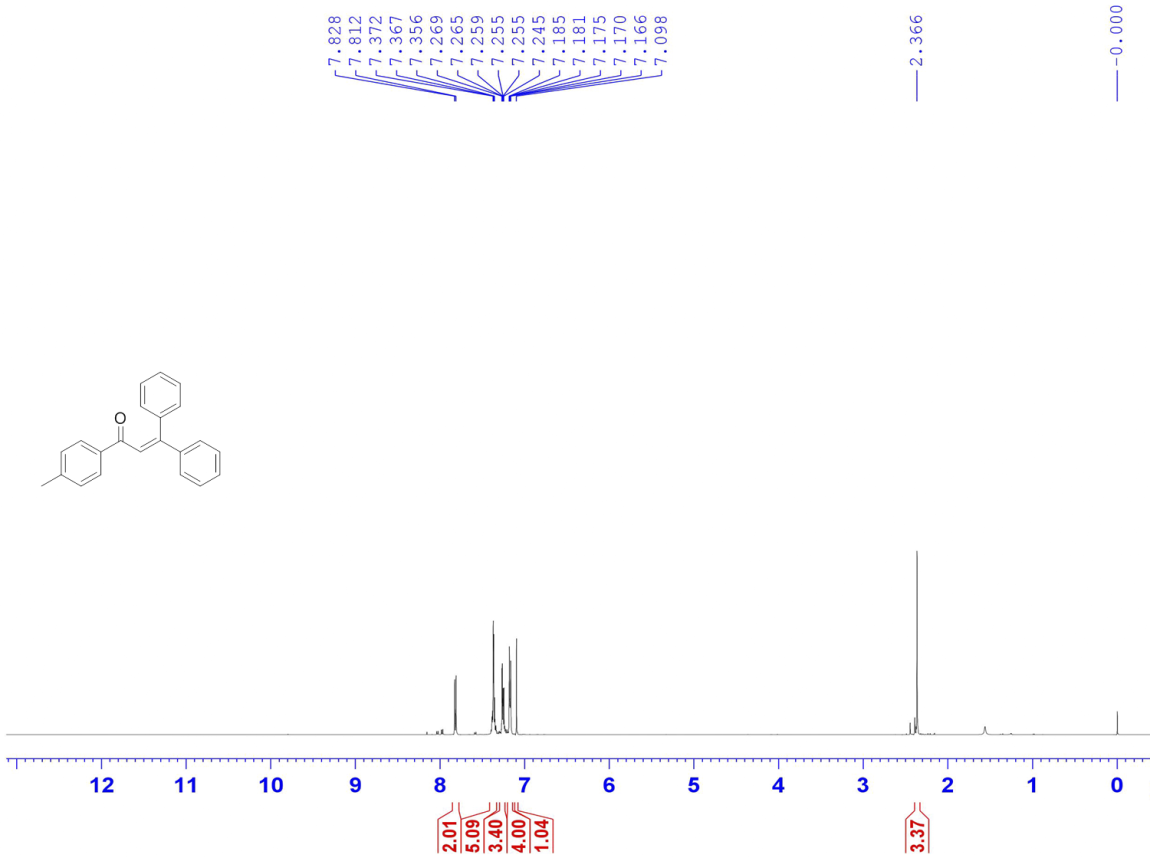


Fig.S7. ^1^H-NMR spectra of 3,3-diphenyl-1-p-tolylprop-2-en-1-one.


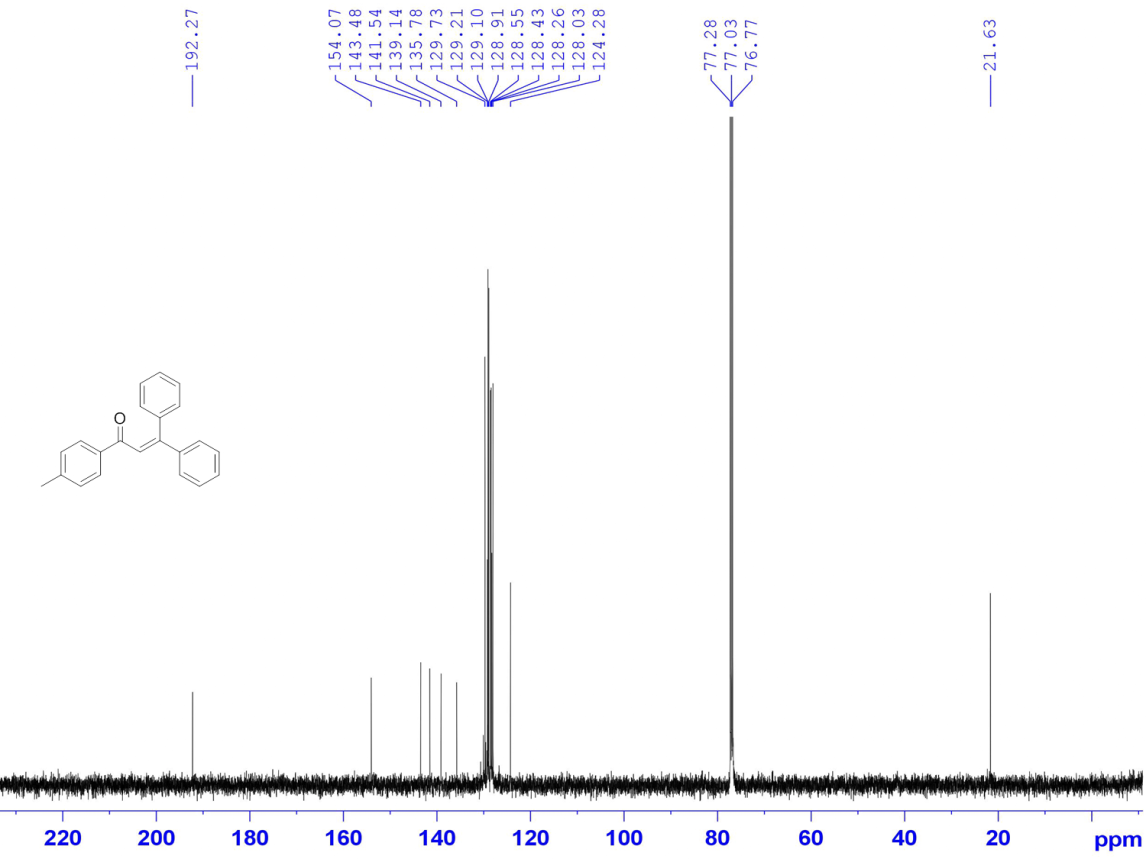


Fig.S8 ^13^C-NMR spectra of 3,3-diphenyl-1-p-tolylprop-2-en-1-one.

**Characterization data for 3,3-diphenyl-1-p-tolylprop-2-en-1-one**

Prepared as shown in the general experimental procedure and purified on silica gel (230-400 mesh or 37-63 μm, ethyl acetate/hexane = 1:15 (v./v.), TLC silica gel 60 F_254_, R_f_ = 0.3): Yellow liquid, 76% yield (68 mg). ^1^H-NMR (500 MHz, CDCl_3_) 2.36 (s, 3H), 7.09 (s, 1H), 7.16-7.18 (m, 4H), 7.24-7.26 (m, 3H), 7.35-7.38 (m, 5H), 7.81 (d, *J* = 8.0 Hz, 2H). ^13^C NMR (CDCl_3_, 125 MHz) δ(ppm) 21.6, 124.2, 128.0, 128.2, 128.4, 128.5, 128.9, 129.1, 129.2, 129.7, 135.7, 139.1, 141.5, 143.4, 154.0, 192.2.


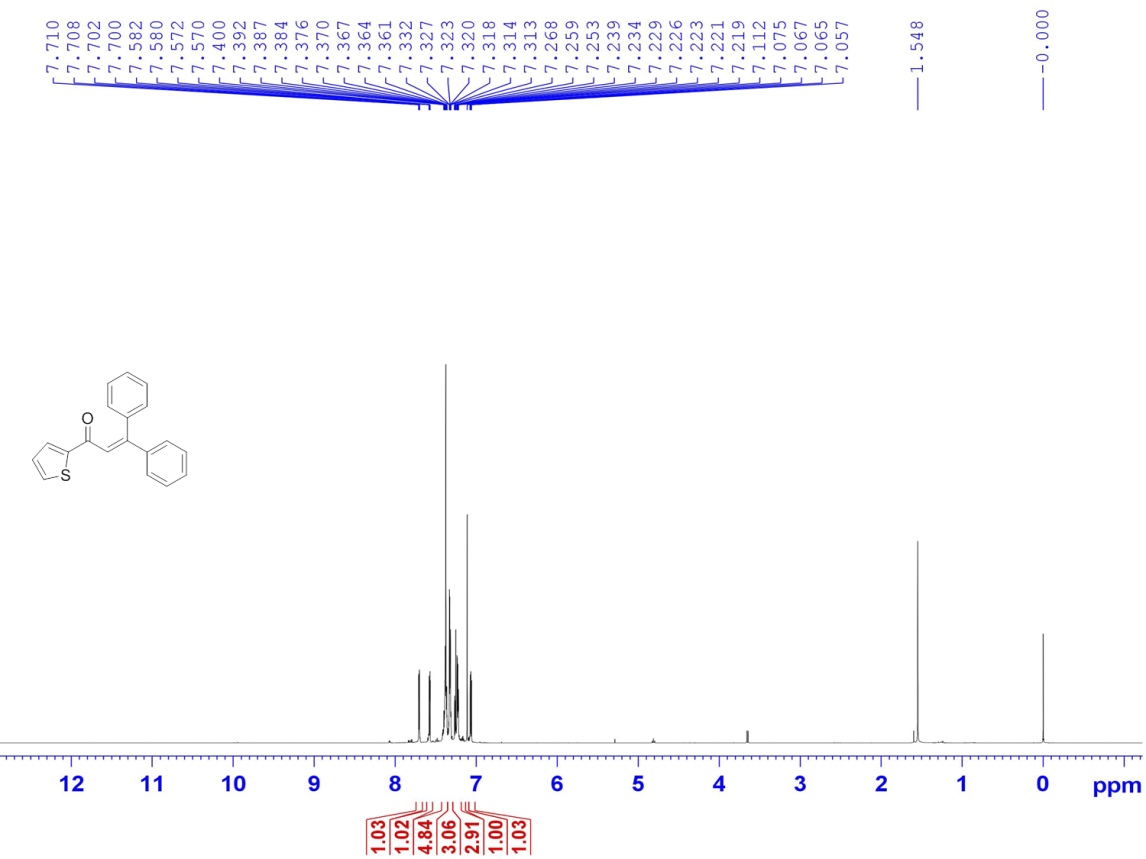


Fig.S9. ^1^H-NMR spectrum of 3,3-diphenyl-1-(thiophen-2-yl)prop-2-en-1-one.


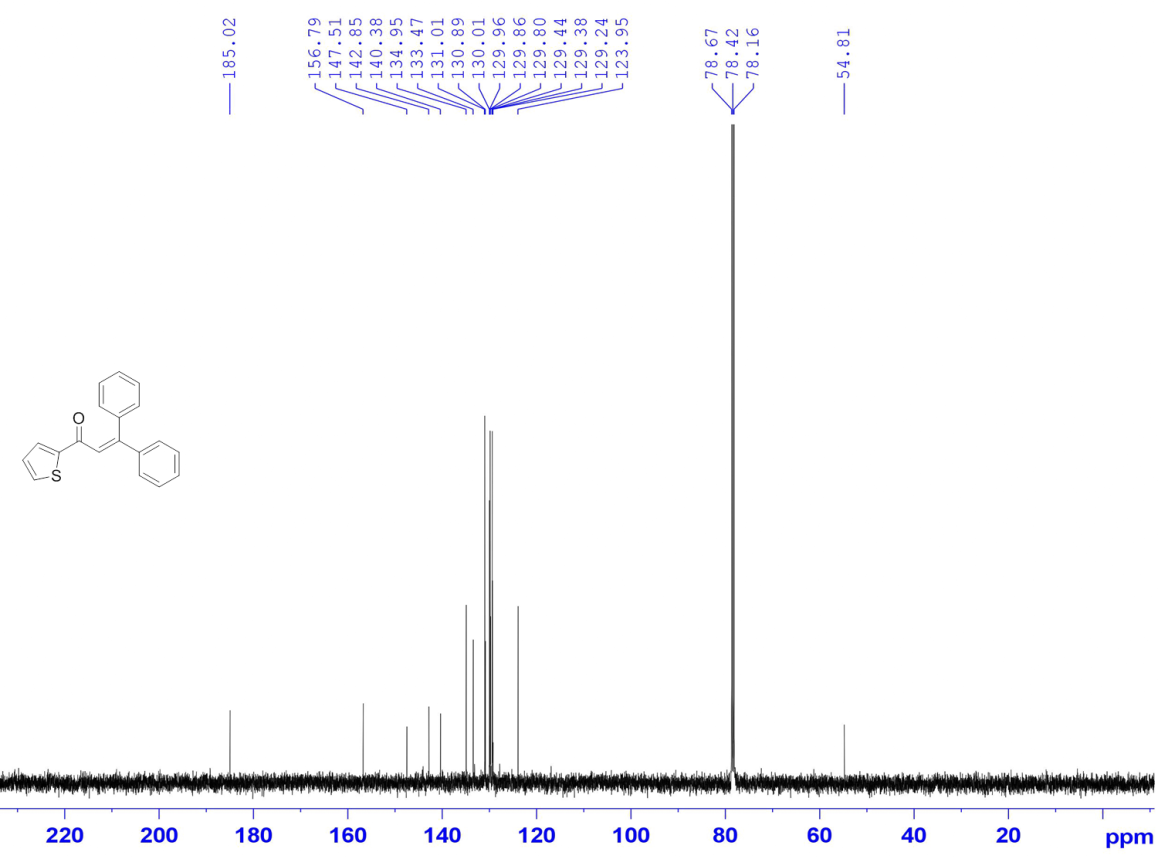


Fig.S10. ^13^C-NMR spectrum of 3,3-diphenyl-1-(thiophen-2-yl)prop-2-en-1-one.

**Characterization data for 3,3-diphenyl-1-(thiophen-2-yl)prop-2-en-1-one**

Prepared as shown in the general experimental procedure and purified on silica gel (230-400 mesh or 37-63 μm, ethyl acetate/hexane = 1:10 (v./v.), TLC silica gel 60 F_254_, R_f_ = 0.45): Yellow liquid, 71% yield (62 mg). ^1^H-NMR (500 MHz, CDCl_3_) 7.05-7.07 (m, 1H), 7.11 (s, 1H), 7.21-7.26 (m, 2H), 7.31-7.33 (m, 3H), 7.36-7.40 (m, 5H), 7.57 (dd, *J* = 5.0 Hz, 1 Hz, 1H), 7.70 (dd, *J* = 4.0 Hz, 1 Hz, 1H). ^13^C NMR (CDCl_3_, 125 MHz) δ(ppm) 123.9, 129.2, 129.3, 129.4, 129.8, 129.8, 129.9, 130.0, 130.8, 131.0, 133.4, 134.9, 140.3, 142.8, 147.5, 156.7, 185.0.


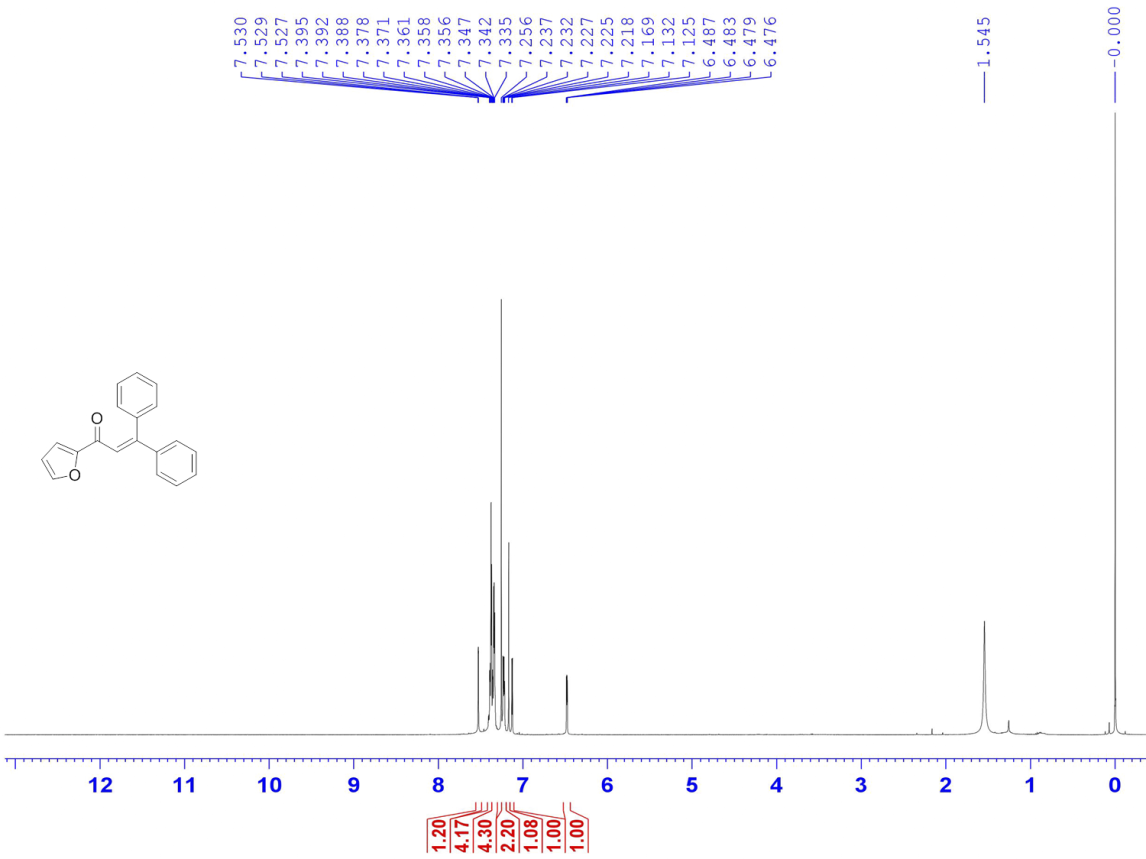


Fig.S11. ^1^H-NMR spectrum of 1-(furan-2-yl)-3,3-diphenylprop-2-en-1-one.


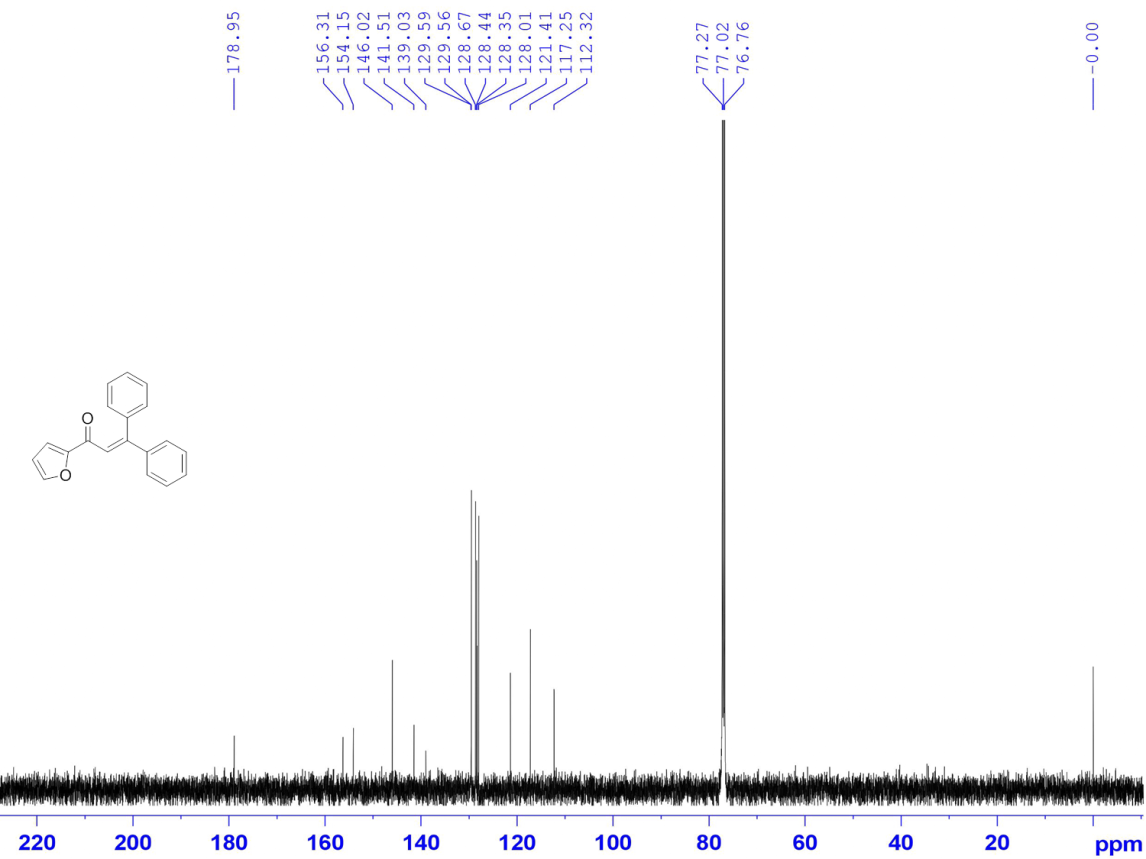


Fig.S12. ^13^C-NMR spectrum of 1-(furan-2-yl)-3,3-diphenylprop-2-en-1-one.

**Characterization data for 1-(furan-2-yl)-3,3-diphenylprop-2-en-1-one**

Prepared as shown in the general experimental procedure and purified on silica gel (230-400 mesh or 37-63 μm, ethyl acetate/hexane = 1:10 (v./v.), TLC silica gel 60 F_254_, R_f_ = 0.4): Yellow liquid, 73% yield (60 mg). ^1^H-NMR (500 MHz, CDCl_3_) 6.47-6.48 (m, 1H), 7.12 (d, *J* = 3.5 Hz, 1H), 7.16 (s, 1H), 7.21-7.23 (m, 2H), 7.33-7.35 (m, 4H), 7.35-7.39 (m, 4H), 7.52-7.53 (m, 1H). ^13^C NMR (CDCl_3_, 125 MHz) δ(ppm) 112.3, 117.2, 121.4, 128.0, 128.3, 128.4, 128.6, 129.5, 129.5, 139.0, 141.5, 146.0, 154.1, 156.3, 178.9.


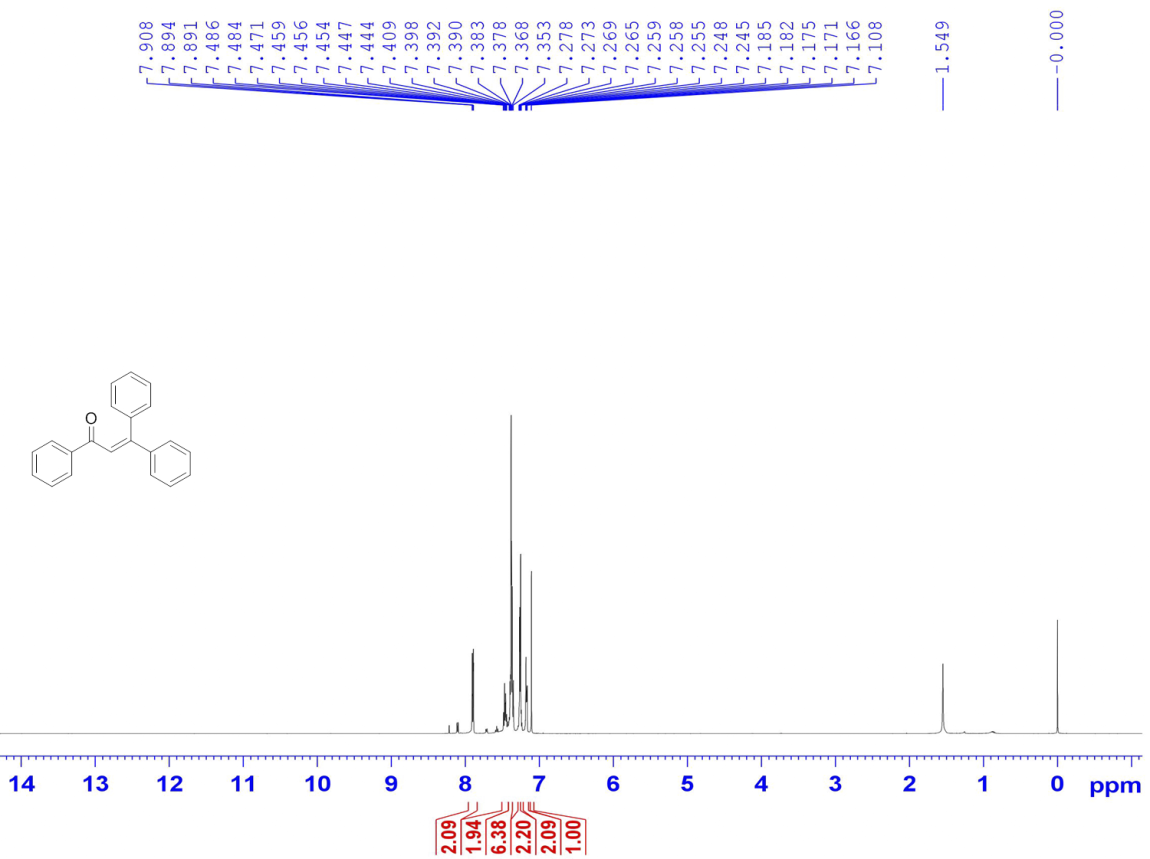


Fig.S13. ^1^H-NMR spectra of 1,3,3-triphenylprop-2-en-1-one.


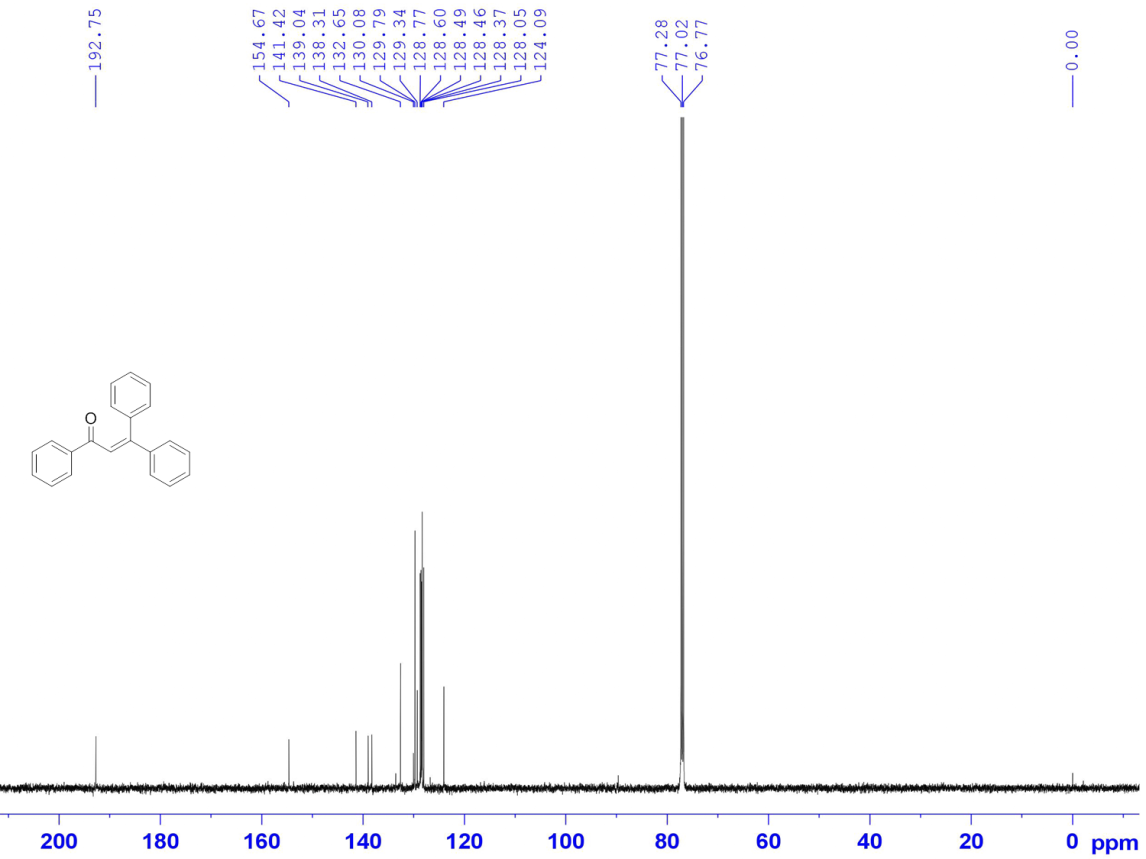


Fig.S14. ^13^C-NMR spectra of 1,3,3-triphenylprop-2-en-1-one.

**Characterization data for 1,3,3-triphenylprop-2-en-1-one**

Prepared as shown in the general experimental procedure and purified on silica gel (230-400 mesh or 37-63 μm, ethyl acetate/hexane = 1:15 (v./v.), TLC silica gel 60 F_254_, R_f_ = 0.4): Yellow liquid, 57% yield (48 mg). ^1^H-NMR (500 MHz, CDCl_3_) 7.10 (s, 1H), 7.16-7.18 (m, 2H), 7.24-7.27 (m, 2H), 7.35-7.40 (m, 7H), 7,44-7.48 (m, 2H), 7.89 (dd, *J* = 8.0 Hz, 1Hz, 2H). ^13^C NMR (CDCl_3_, 125 MHz) δ(ppm) 124.0, 128.0, 128.3, 128.4, 128.4, 128.6, 128.7, 129.3, 129.7, 130.0, 132.6, 138.3, 139.0, 141.4, 154.6, 192.7.


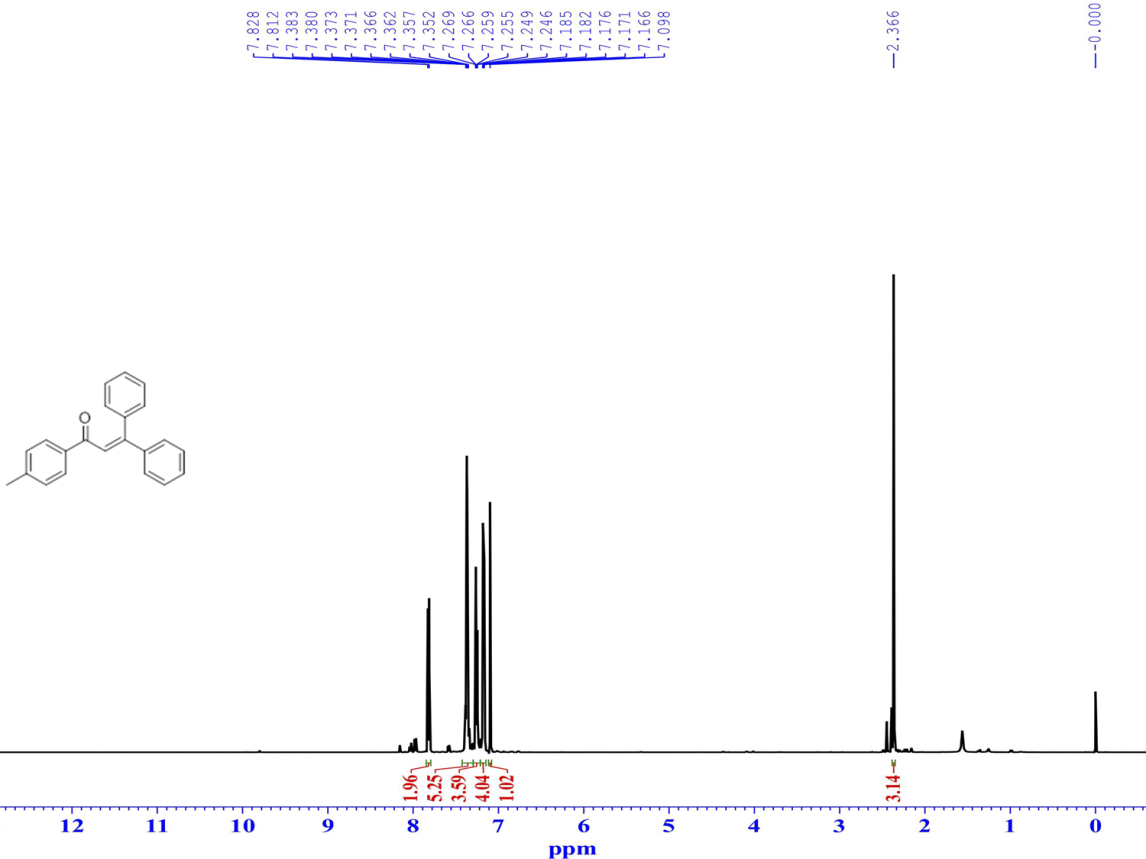


Fig.S15. ^1^H-NMR spectra of 3,3-diphenyl-1-p-tolylprop-2-en-1-one.


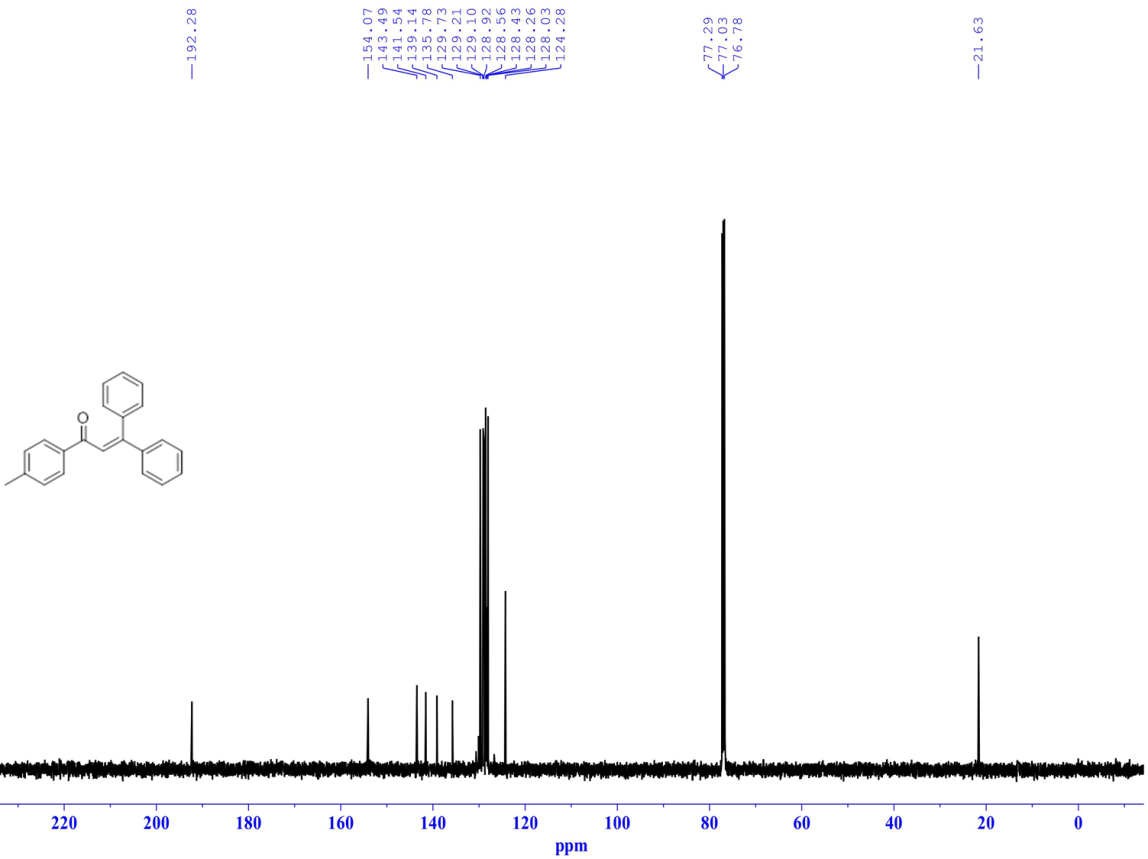


Fig.S16. ^13^C-NMR spectra of 3,3-diphenyl-1-p-tolylprop-2-en-1-one.

**Characterization data for 3,3-diphenyl-1-p-tolylprop-2-en-1-one**

Prepared as shown in the general experimental procedure and purified on silica gel (230-400 mesh or 37-63 μm, ethyl acetate/hexane = 1:15 (v./v.), TLC silica gel 60 F_254_, R_f_ = 0.3): Yellow liquid, 53% yield (47 mg). ^1^H-NMR (500 MHz, CDCl_3_) 2.36 (s, 3H), 7.09 (s, 1H), 7.16-7.18 (m, 4H), 7.24-7.29 (m, 3H), 7.34-7.38 (m, 5H), 7.81-7.82 (d, *J* = 8.0 Hz, 2H). ^13^C NMR (CDCl_3_, 125 MHz) δ(ppm) 21.6, 124.2, 128.0, 128.2, 128.4, 128.5, 128.9, 129.1, 129.2, 129.7, 135.7, 139.1, 141.5, 143.4, 154.0, 192.2.


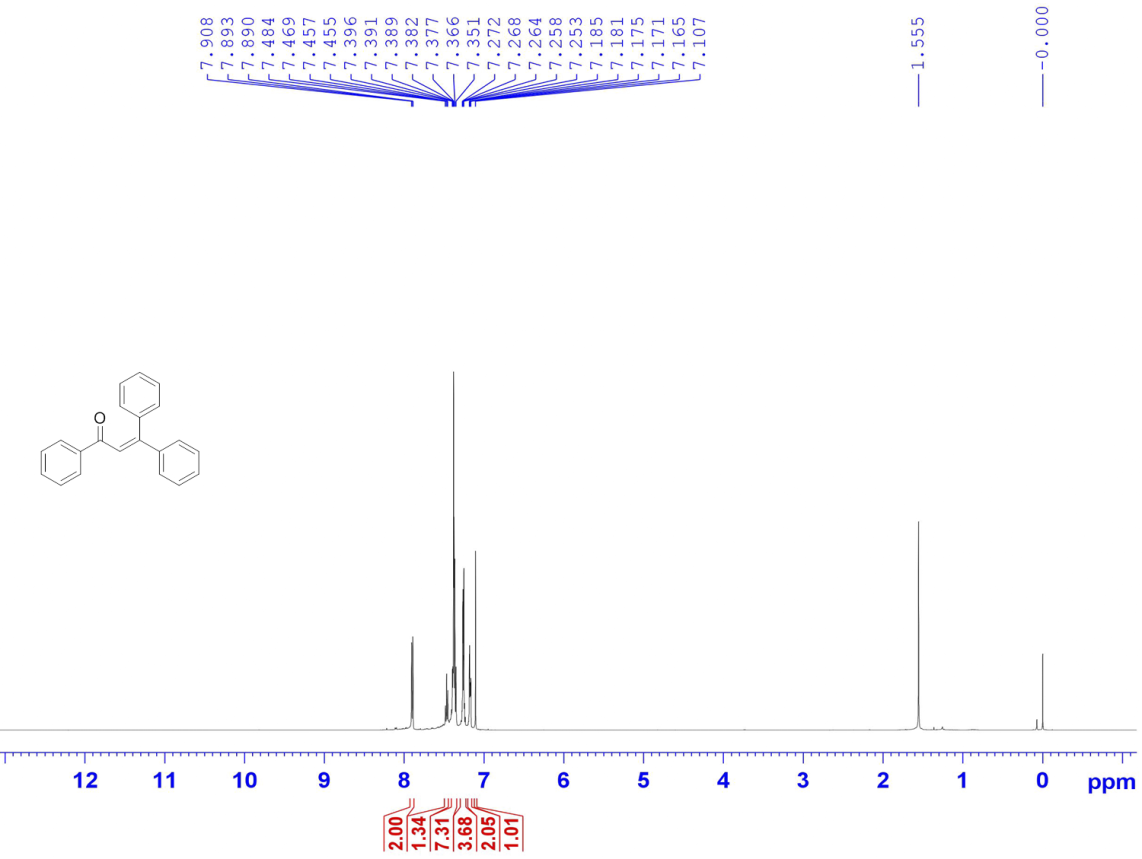


Fig.S17. ^1^H-NMR spectra of 1,3,3-triphenylprop-2-en-1-one.


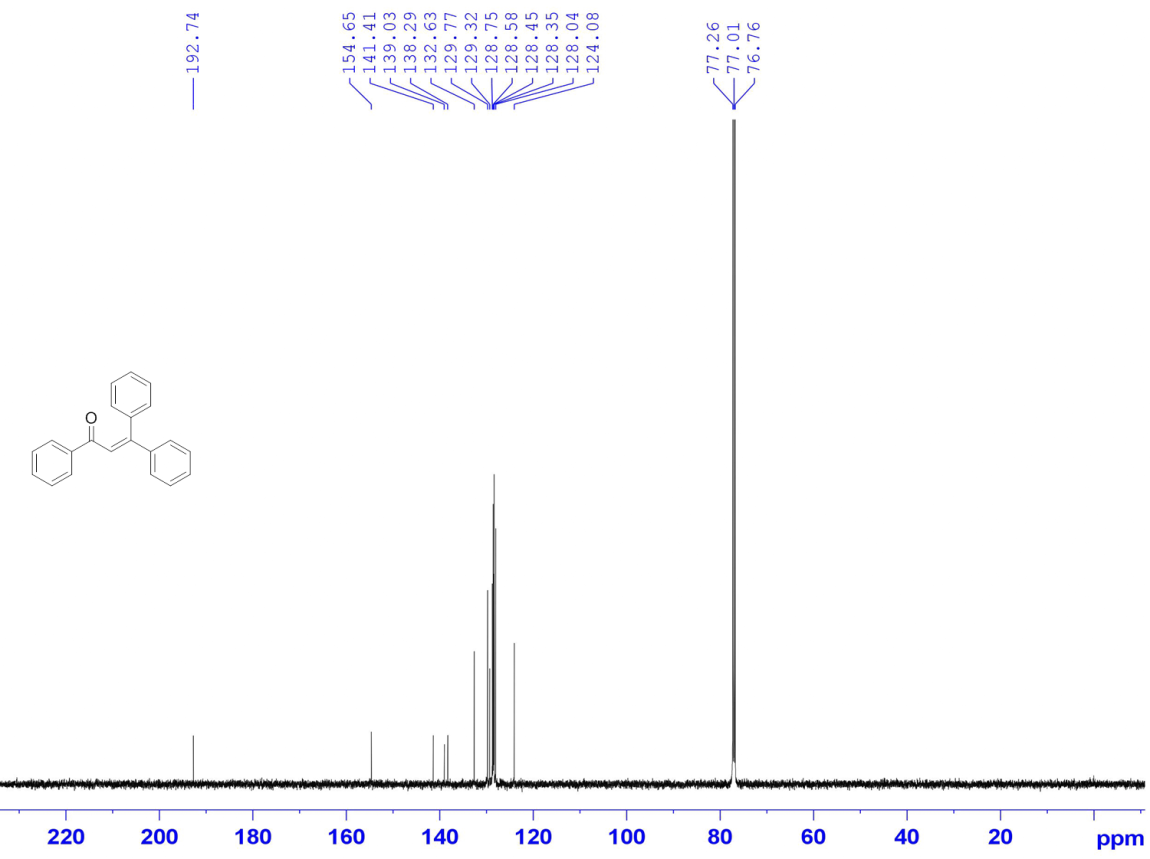


Fig.S18. ^13^C-NMR spectra of 1,3,3-triphenylprop-2-en-1-one.

**Characterization data for 1,3,3-triphenylprop-2-en-1-one**

Prepared as shown in the general experimental procedure and purified on silica gel (230-400 mesh or 37-63 μm, ethyl acetate/hexane = 1:15 (v./v.), TLC silica gel 60 F_254_, R_f_ = 0.4): Yellow liquid, 51% yield (43 mg). ^1^H-NMR (500 MHz, CDCl_3_) 7.10 (s, 1H), 7.16-7.18, m, 2H), 7.25-7.27 (m, 3H), 7.35-7.39 (m, 7H), 7.45-7.48 (m, 1H), 7.89 (dd, *J* = 8.0 Hz, 1Hz 2H). ^13^C NMR (CDCl_3_, 125 MHz) δ(ppm) 124.0, 128.0, 128.3, 128.4, 128.5, 128.7, 129.3, 129.7, 132.6, 138.2, 139.0, 141.4, 154.6, 192.7.


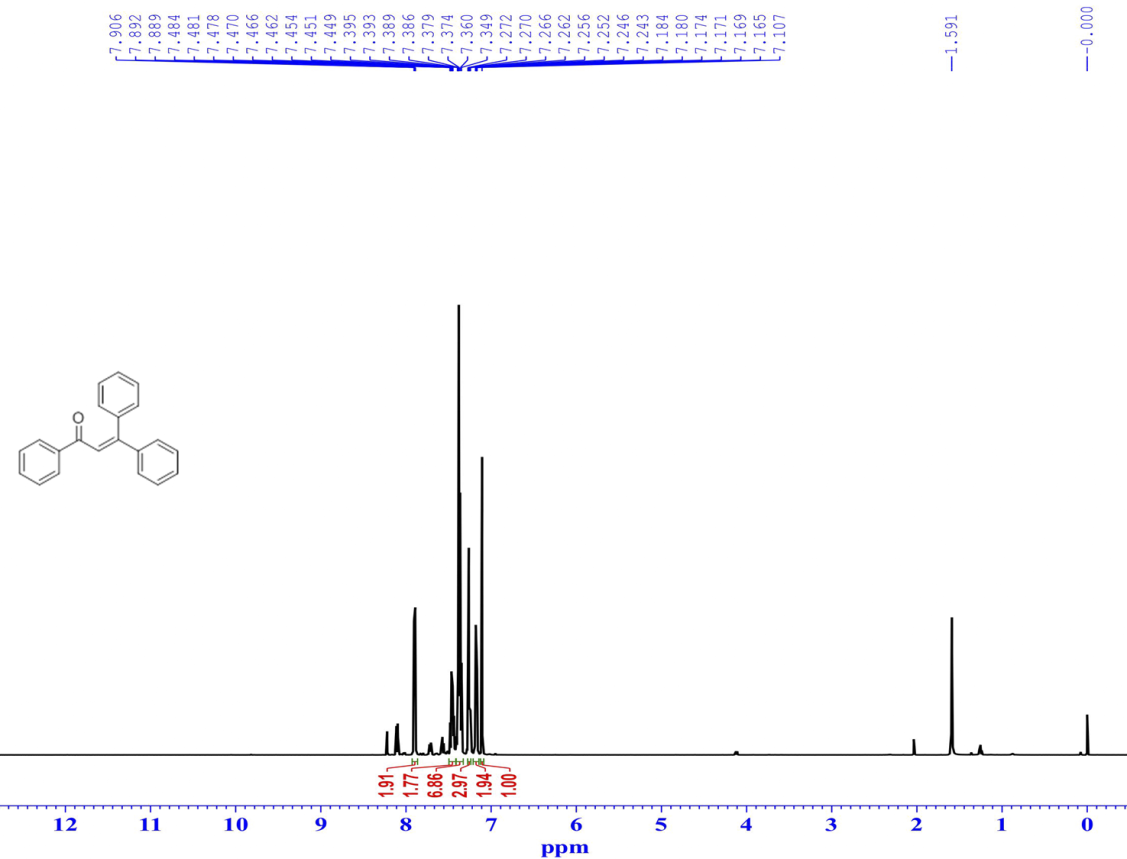


Fig.S19. ^1^H-NMR spectra of 1,3,3-triphenylprop-2-en-1-one.


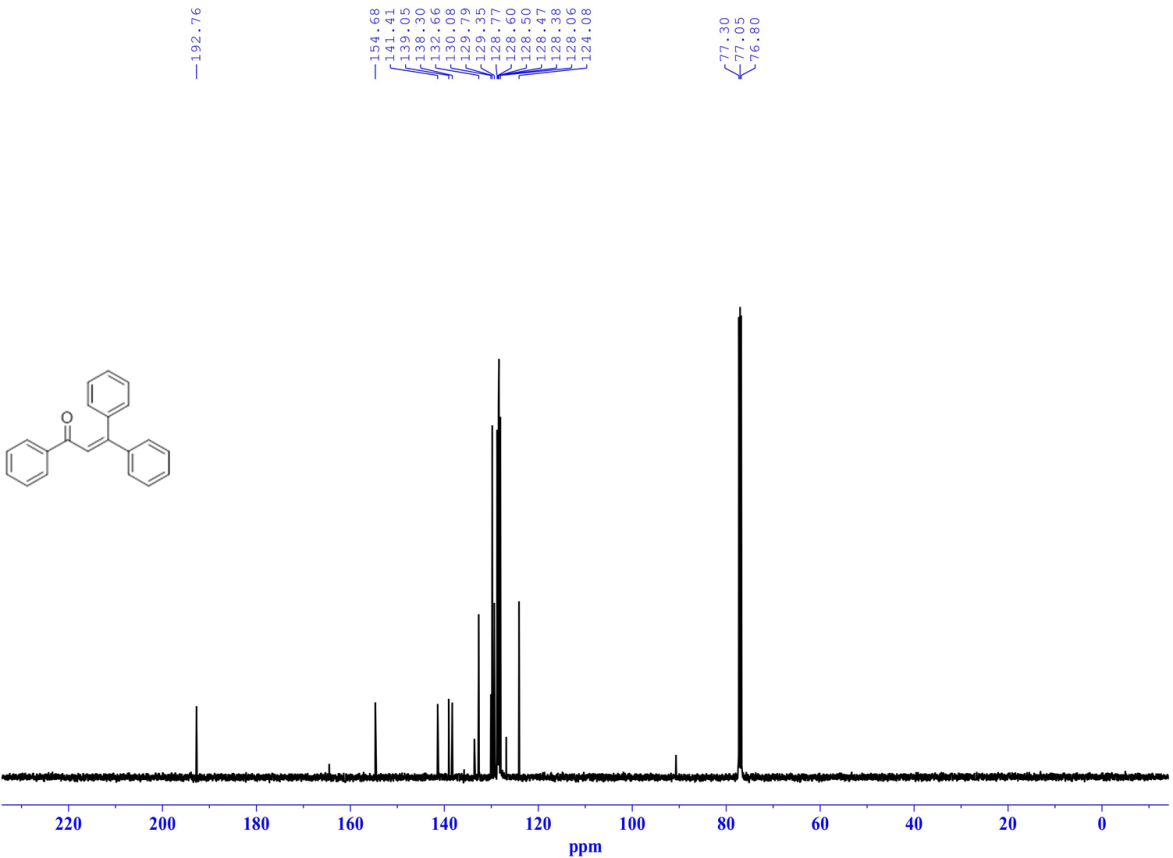


Fig.S20. ^13^C-NMR spectra of 1,3,3-triphenylprop-2-en-1-one.

**Characterization data for 1,3,3-triphenylprop-2-en-1-one**

Prepared as shown in the general experimental procedure and purified on silica gel (230-400 mesh or 37-63 μm, ethyl acetate/hexane = 1:15 (v./v.), TLC silica gel 60 F_254_, R_f_ = 0.35): Light yellow liquid, 71% yield (60 mg). ^1^H-NMR (500 MHz, CDCl_3_) 7.10 (s, 1H), 7.16-7.18 (m, 2H), 7.24-7.27 (m, 3H), 7.34-7.39 (m, 7H), 7.44-7.48 (m, 1H), 7.89-7.90 (d, *J* = 5.0 Hz, 2H). ^13^C NMR (CDCl_3_, 125 MHz) δ(ppm) 124.0, 128.0, 128.3, 128.4, 128.5, 128.6, 128.7, 129.3, 129.7, 130.0, 132.6, 138.3, 139.0, 141.4, 154.6, 192.7.


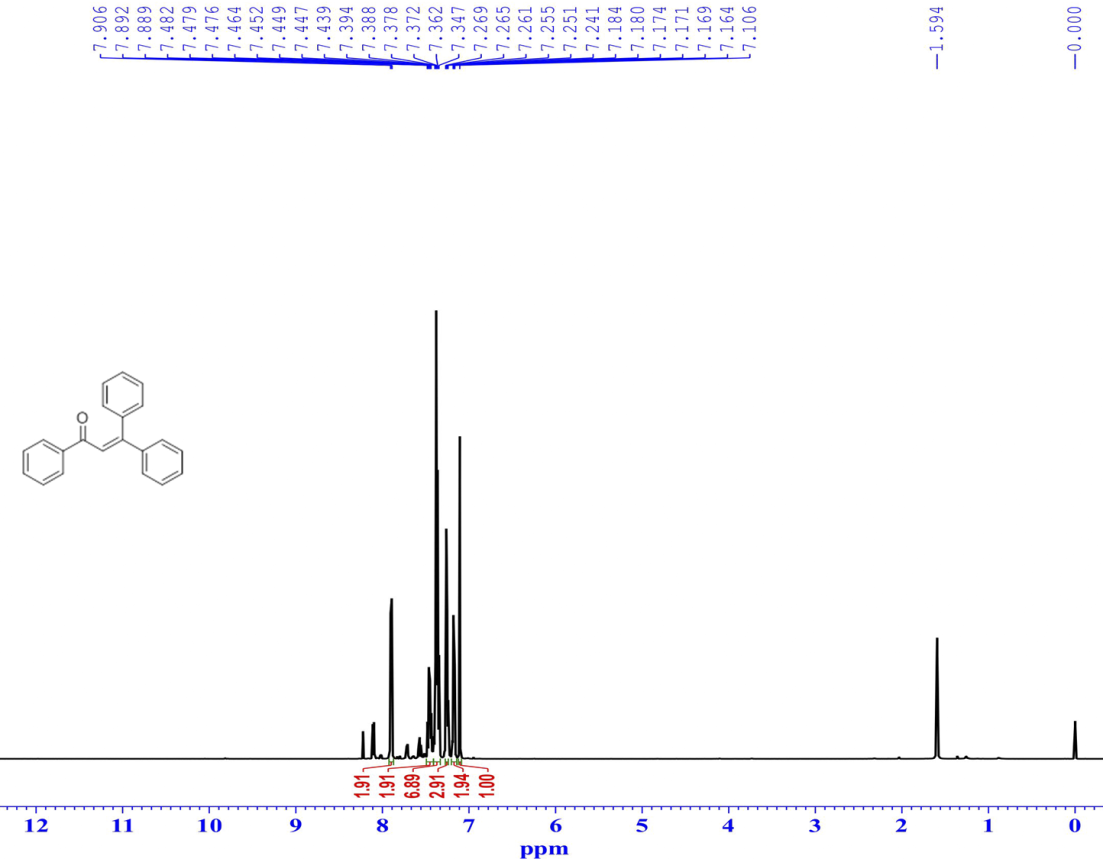


Fig.S21. ^1^H-NMR spectra of 1,3,3-triphenylprop-2-en-1-one.


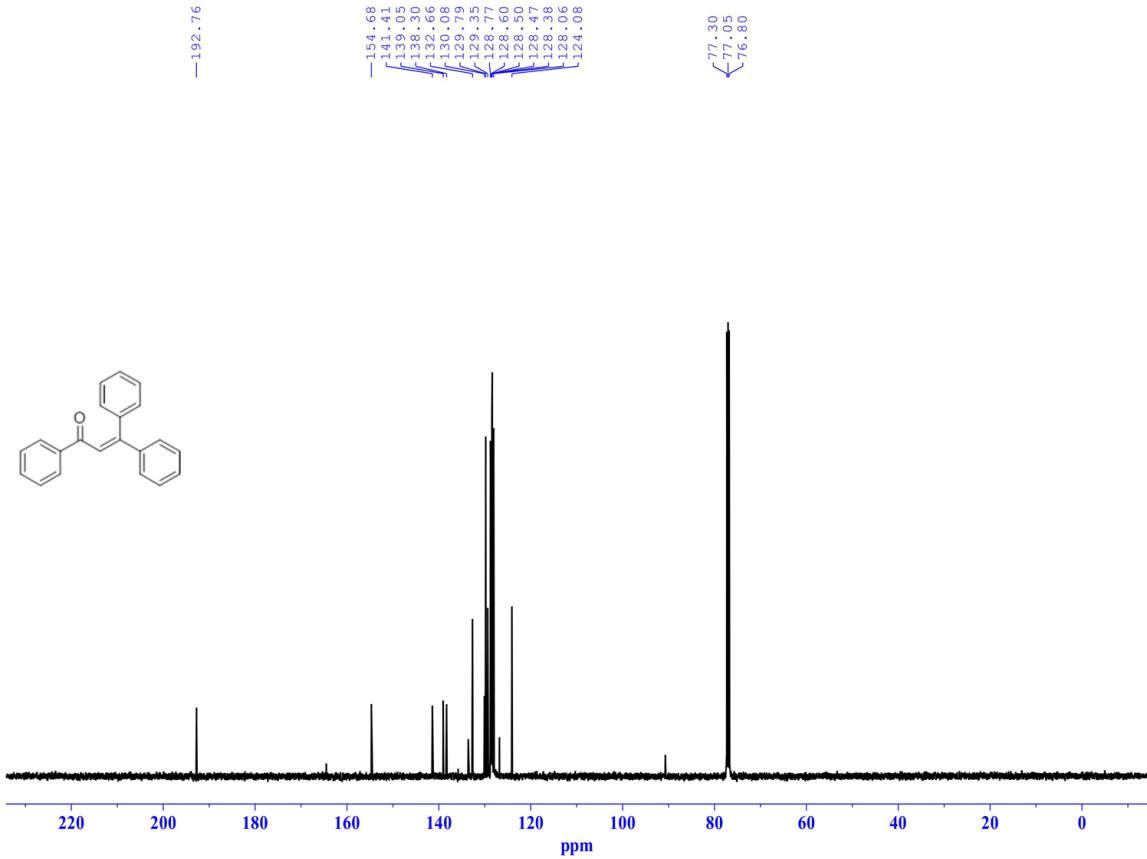


Fig.S22. ^13^C-NMR spectra of 1,3,3-triphenylprop-2-en-1-one.

**Characterization data for 1,3,3-triphenylprop-2-en-1-one**

Prepared as shown in the general experimental procedure and purified on silica gel (230-400 mesh or 37-63 μm, ethyl acetate/hexane = 1:15 (v./v.), TLC silica gel 60 F_254_, R_f_ = 0.3): Light yellow liquid, 69% yield (59 mg). ^1^H-NMR (500 MHz, CDCl_3_) 7.10 (s, 1H), 7.16-7.18 (m, 2H), 7.24-7.26 (m, 3H), 7.34-7.39 (m, 7H), 7.43-7.48 (m, 1H), 7.89-7.90 (d, *J* = 5.0 Hz, 2H). ^13^C NMR (CDCl_3_, 125 MHz) δ(ppm) 124.0, 128.0, 128.3, 128.4, 128.5, 128.6, 128.7, 129.3, 129.7, 130.0, 132.6, 138.3, 139.0, 141.4, 154.6, 192.7.


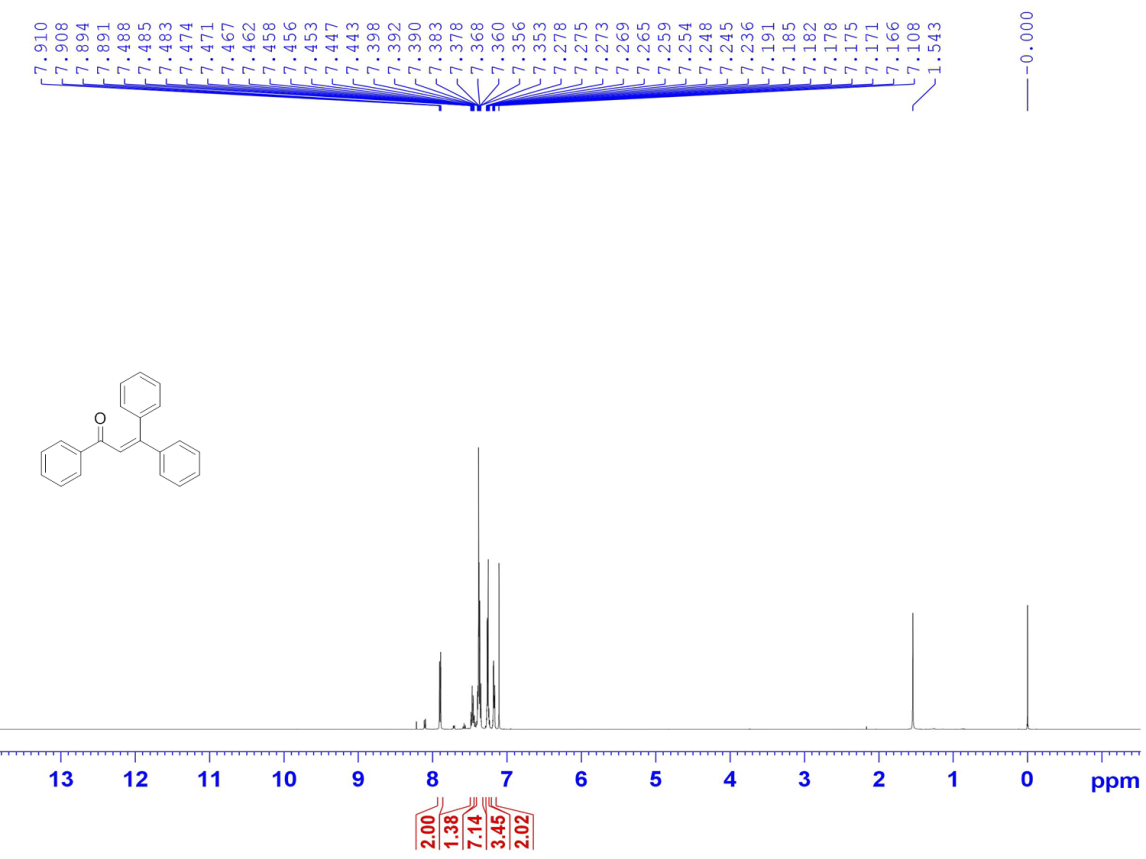


Fig.S23. ^1^H-NMR spectra of 1,3,3-triphenylprop-2-en-1-one.


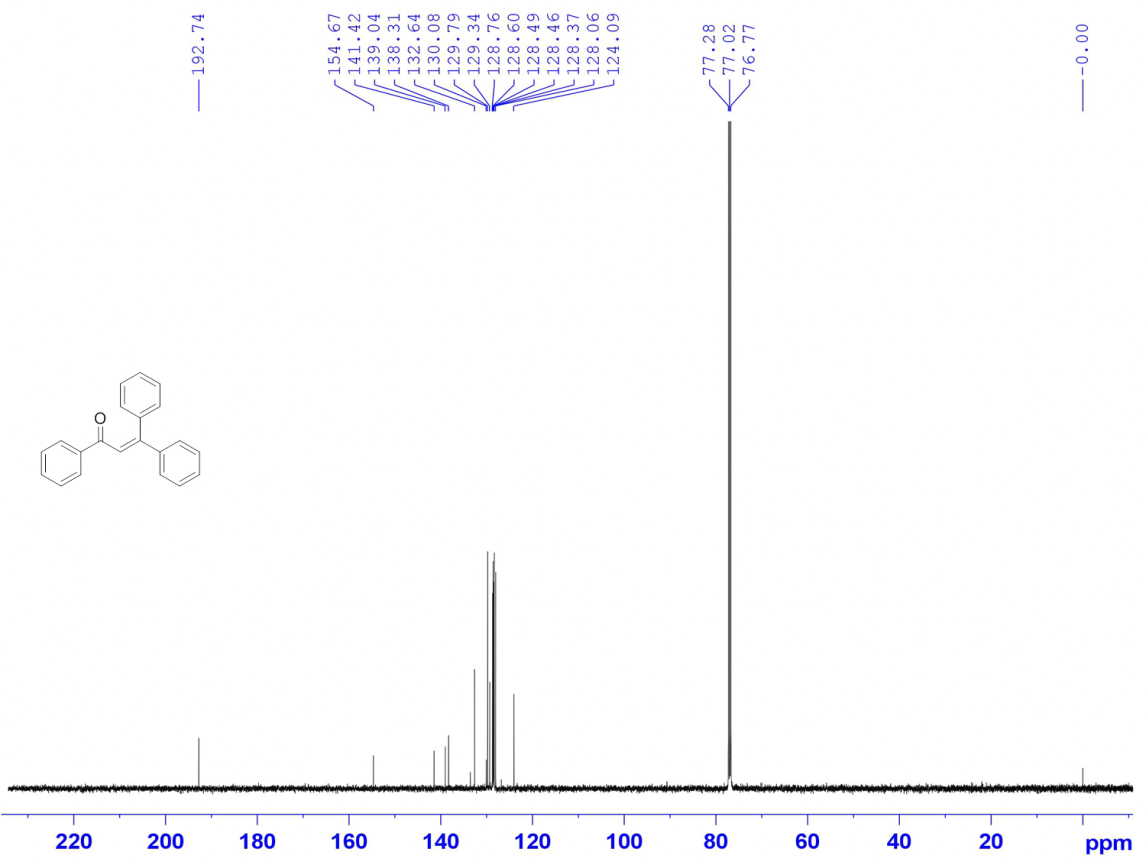


Fig.S24. ^13^C-NMR spectra of 1,3,3-triphenylprop-2-en-1-one.

**Characterization data for 1,3,3-triphenylprop-2-en-1-one**

Prepared as shown in the general experimental procedure and purified on silica gel (230-400 mesh or 37-63 μm, ethyl acetate/hexane = 1:15 (v./v.), TLC silica gel 60 F_254_, R_f_ = 0.4): Yellow liquid, 66% yield (56 mg). ^1^H-NMR (500 MHz, CDCl_3_) 7.10 (s, 1H), 7.16-7.19 (m, 2H), 7.23-7.27 (m, 3H), 7.35-7.39 (m, 7H), 7.44-7.48 (m, 1H), 7.89 (dd, *J* = 8.0 Hz, 1.0 Hz, 2H). ^13^C NMR (CDCl_3_, 125 MHz) δ(ppm) 124.0, 128.0, 128.3, 128.4, 128.4, 128.6, 128.7, 129.3, 129.7, 130.0, 132.6, 138.3, 139.0, 141.4, 154.6, 192.7.
